# Differential expression of pluripotent and germ cell markers in ovarian surface epithelium according to age in female mice

**DOI:** 10.1186/1477-7827-12-113

**Published:** 2014-11-24

**Authors:** Bo Sun Joo, In Kook Jung, Min Jung Park, Jong Kil Joo, Ki Hyung Kim, Kyu-Sup Lee

**Affiliations:** Research Center for Anti-Aging Technology Development, Pusan National University, Busan, Korea; Department of Obstetrics and Gynecology, Medical Research Institute, Pusan National University School of Medicine, Busan, Korea

**Keywords:** Putative ovarian stem cells (OSCs), Ovarian surface epithelium (OSE), Pluripotent and germ cell markers, Female age

## Abstract

**Background:**

Many studies have proposed that putative ovarian stem cells (OSCs) derived from the ovarian surface epithelium (OSE) layer of adult mammalian ovaries can produce oocytes. Few studies have reported that ovaries of aged mammalian females including mice and women possess rare premeiotic germ cells that can generate oocytes. However, no studies have reported the changes of OSCs according to the age of the female. Therefore, this study evaluated pluripotent and germ cell marker expression in the intact ovary, scraped OSE, and postcultured OSE according to age in female mice.

**Methods:**

C57BL/6 female mice of 2 age groups (6–8 and 28–31 weeks) were superovulated by injection with 5 IU equine chorionic gonadotropin (eCG). Both ovaries were removed after 48 hours and scrapped to obtain OSE. Gene expressions of pluripotent (Oct-4, Sox-2, Nanog) and germ cell markers (c-Kit, GDF-9, and VASA) were evaluated by RT-PCR. VASA and GDF-9 were immune-localized in oocyte-like structures.

**Results:**

Expressions of germ cell markers in the intact ovary were significantly decreased in aged females, whereas expressions of pluripotent markers were not detected, regardless of age. Scraped OSE expression of all pluripotent and germ cell markers, except for c-Kit, was similar between both age groups. Three weeks postcultured OSE had significantly decreased expression of GDF-9 and VASA , but not c-Kit, in old mice, as compared to young mice; however there was no difference in the expression of other genes. The number of positively stained Oct-4 by immunohistochemistry in postcultured OSE was 2.5 times higher in young mice than aged mice. Oocyte-like structure was spontaneously produced in postcultured OSE. However, while that of young mice revealed a prominent nucleus, zona pellucida-like structure and cytoplasmic organelles, these features were not observed in old mice.

**Conclusions:**

These results show that aged female mice have putative OSCs in OSE, but their differentiation potential, as well as the number of OSCs differs from those of young mice.

## Background

Advancing female age is closely associated with a decrease in the number and quality of ovulated oocytes. Various methods including the activation of ovarian angiogenesis have been attempted to improve oocyte quality in the aged female [1–4]. However, it remains a challenging problem in the treatment of infertility.

Since Tilly’s group first reported the existence of proliferative germline stem cells that sustain oocyte and follicle production in the postnatal mouse ovary [5], many studies have subsequently demonstrated that putative ovarian stem cells (OSCs) can be successfully isolated from the ovarian surface epithelium (OSE) of the neonatal and adult mammalian ovary, including mice and human [6–8]. This concept has challenged the traditional central dogma of mammalian reproductive biology that female are born with a finite and non-renewable pool of oocyte-containing follicles [9]. Nevertheless, these findings suggested that postnatal oocyte renewal using OSE-derived OSCs will be helpful to better management and understanding of menopause, reproductive disease, and infertility associated with old age, poor response, or premenopause ovarian failure (POF).

OSEs are epithelial cells covering the ovaries and they are relatively less differentiated, uncommitted layer of cells that express both epithelial and mesenchymal markers [10, 11]. Recently, Parte *et al*. introduced a new concept that OSE-derived OSCs possess of 2 distinct stem cell populations including the pluripotent very small embryonic like stem cells (VSELs) and their immediate descendent ‘progenitor’ ovarian germ stem cells (OGSCs) in most adult mammals, including mice, rabbits, sheep, monkeys, and women [11, 12].

Several recent studies have shown successful postnatal oocyte renewal from OSCs derived from the OSE. Zou *et al.* reported the production of offspring after transplantation of a germline stem cell line derived from the neonatal mouse ovary into ovaries of infertile mice [13]. Niikura *et al*. showed that aged mouse ovaries possess a rare population of premeiotic germ cells that retain the capacity to form oocytes on exposure to a young host environment [14]. White *et al*. reported that ovaries from women of reproductive-age also possess rare mitotically active germ cells that can be propagated *in vitro*, as well as generate oocytes *in vitro* and *in vivo*[15]. These results suggested that OSE-derived OSCs can produce primordial germ cells and oocytes if appropriate conditions are provided. Therefore, if OSCs existing in the aged ovary could continuously generate good quality oocytes, it may provide a basis of effective treatment for age-related decline of fertility in females. However, few studies have reported that ovaries of aged female, including mice and women, possess rare premeiotic germ cells that can generate oocytes [11, 14, 16]. Accordingly, in the current study model we examined the change of pluripotent and germ cell markers expression in the intact ovary, scraped OSE cells, and postcultured OSE cells according to female mice age.

## Methods

### Animals

C57BL/6 inbred mice were purchased from Korea Experimental Animal Center (Daegu, South Korea). The mice were maintained on a continuous cycle of lights on at 7:00 AM, and off at 7:00 PM, with food and water available *ad libitum* under specified pathogen free (SPF) condition. The food was provided as pellets (Global Rodent Diet, Harlan Lab., Indianapolis, USA) and water was sterilized and provided in water bottles. The room temperature was maintained at 21 ± 2°C and the relative humidity at 55 ± 10%.

This study was approved from the Institutional Review Board of Pusan National University Hospital, Korea. Female mice of two age groups (6–8 and 28–31 weeks) were injected intraperitoneally with 5 IU of eCG (Sigma, St. Louis, MO, USA). eCG was injected for two purposes: one is to synchronize the estrus cycle and the other is to increase pluripotent stem cell activity. Bhartiya *et al*. showed that eCG treatment resulted in increased pluripotent stem cell activity, neo-oogenesis and PF assembly in the adult mouse ovaries. Especially, two days after eCG treatment, OSE exhibited extensive proliferation [12]. Forty-eight hours after eCG injection, the mice were sacrificed by cervical dislocation and both ovaries were collected.

### Scraping of ovarian surface epithelium (OSE) and *in vitro*culture of putative ovarian stem cells (OSCs)

OSCs were retrieved from OSE. Both ovaries were gently rinsed several times in Dulbecco’s phosphate-buffered saline (DPBS; Invitrogen, Carlsbad, CA, USA) containing antibiotics (penicillin 100 U/mL, streptomycin 100 mg/mL; Invitrogen) at ambient temperature and kept in serum-free plain and preincubated Dulbecco's Modified Eagle Medium**/**Ham's Nutrient Mixture F-12 (DMEM/F12; Gibco BRL, Grand Island, NY, USA) before scraping of OSE. One intact ovary was provided for reverse-transcription polymerase chain reaction (RT-PCR). The surface of the remaining intact ovary was gently scraped several times in the aseptic laminar flow hood with a sterile disposable surgical scalpel (Swann-Morton, Sheffield, United Kingdom) into plain DMEM/F12 in a 60 mm dish at 37°C on a preheated tray. OSE was easily detached from the ovarian surface and centrifuged to retrieve a scraped suspension of cells. The suspension of scraped OSE cells were transferred to a 15 mL centrifuge tube and spun at 1,000 g for 10 minutes at room temperature. The pellet was suspended in fresh medium and cultured in DMEM/F12 supplemented with 20% fetal bovine serum (FBS; Invitrogen) and antibiotics (Invitrogen) in a 5% CO_2_ incubator at 37°C for 3 weeks. The culture medium was replaced with fresh medium every 2 days. Cultures were carefully monitored daily under a heated stage inverted microscope (ECLIPSE 2000-S, Nikon, Tokyo, Japan) equipped with a digital sight camera (Nikon**,** Tokyo, Japan). The intact ovary, the scraped OSE cells and the postcultured OSE cells were utilized for reverse-transcription polymerase chain reaction (RT-PCR) and immunohistochemistry.

### Characterization studies

Attached cell cultures including oocyte-like structures were washed with DPBS and enzymatically detached from the plates with a trypsin-EDTA solution (Sigma) for 5 minutes at 37°C. The cell suspension was then centrifuged at 1,000 g for 10 minutes and the pellet was resuspended in Trizol (Invitrogen) and stored at −80°C for RNA extraction. For immunohistochemical analyses, OSCs cultured for 3 weeks (postcultured OSCs) were fixed in a 4% paraformaldehyde (PFA) solution (Sigma) in DPBS for 10 minutes. The cells were air-dried, washed twice with PBS, air-dried again, and stored at 4°C until further use. All characterization studies were carried out in at least 10 mice per each age group.

### RNA preparation and RT-PCR

Both ovaries per mouse were carefully collected. One intact ovary was utilized for RT-PCR. The surface of the remaining intact ovary was gently scraped.

Total RNA was extracted using Trizol reagent (Invitrogen) according to the manufacturer’s protocol. Complementary DNA (cDNA) was synthesized from 1 μg of total RNA with AMV Reverse Transcriptase (Promega, Madison, WI, USA) using a random hexamer (Bioneer, Daejeon, Korea) at 42°C for 1 hour. Each cDNA was subjected to polymerase chain reaction (PCR) amplification using gene-specific primers (Table 1). Pluripotent markers were analyzed for Oct-4, Sox-2 and Nanog transcript markers. Germ cell markers were analyzed for c-Kit, GDF-9 and VASA transcript markers. GAPDH expression was used as an internal control in RT-PCR. PCR products were visualized by 1.2% agarose gel electrophoresis. The PCR bands were quantified and normalized relative to the control band with Image J (National Institutes of Health Image software, version 1.35d, Bethesda, MD, USA). Data were representative of at least 3 independent experiments.

**Table 1:** **Primers sequences used for PCR amplification and conditions**

Gene	Sequence	Tm (°C)	Cycles	Product size (bp)
Oct-4	FW: AGCACGAGTGGAAAGCAACTC	57	38	497
	RV: CAAGCTGATTGGCGATGTGAG			
Sox-2	FW: ACGCTCATGAAGAAGGATAA	58	42	330
	RV: GTAGGACATGCTGTAGGTGG			
Nanog	FW: TACCTCAGCCTCCAGCAGAT	56	42	460
	RV: CACCTCCAA ATCACTGGCAG			
c-Kit	FW: GTCATTGTTGGCTACGAGAT	57	28	142
	RV: AACACGAGGTCATCCACTAT			
GDF-9	FW: TTGCTGTTGCCTGTAGATGG	57	28	204
	RV: GAAGAGCCGGACGGTATTGT			
VASA	FW: GCTCAA ACAGGGTCTGGGAAG	58	35	145
	RW: GGTTGATCAGTTCTCGAGTTC			
GAPDH	FW: ACCACAGTCCATGCCATCAC	57	25	452
	RV: TCCACCACCCTGTTGCTGTA			

### Immunohistochemistry

The GDF-9 (ab93892), VASA (ab13840), and Oct-4 (ab18976) primary antibodies used in this study were purchased from Abcam (Cambridge, MA, USA). Postcultured OSE cells from young and aged mice were grown on glass coverslips and were then fixed in 4% paraformaldehyde (PFA) in PBS, for 10 minutes at room temperature (RT). Coverslips were washed three times with PBS, and stained with anti-GDF-9, VASA, and Oct-4 primary antibody (1:100 dilutions in 1% BSA/PBS solution, overnight at 4°C). The remaining steps were performed according to instructions supplied with the GDF-9, VASA and Oct-4 antibodies. Briefly, after overnight incubation with respective primary antibodies, cells were washed thrice with PBS and samples were incubated with biotinylated-conjugated secondary antibody and HRP coupled to streptavidin-conjugated antibody (Zymed Laboratories-Invitrogen, San Francisco, CA, USA) for 15 minutes at room temperature and washed thrice with PBS. Antibody binding was visualized using 3,3-diaminobenzidine (DAB), counterstained with Mayer’s hematoxylin (Sigma), and mounted with Histomount solution (Invitrogen). The immune-stained Oct-4-positive cells in postcultured OSE were counted under a microscope. Data was representative of at least 2 independent experiments.

### Statistics

Statistical analysis was done with the SPSS program (version 12.0) and all data were presented as a mean ± SD. Comparative expression according to female age was analyzed by student t-test. *P <;* 0.05 was considered to be statistically significant.

## Results

**Figure 1:**
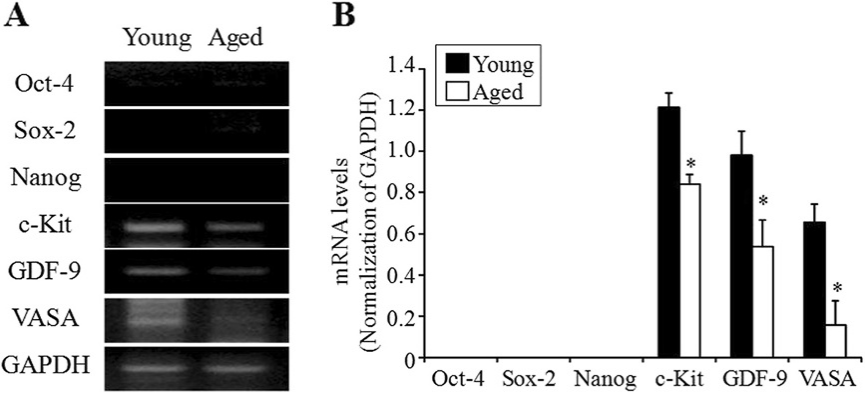
**RT-PCR analysis of pluripotent and germ cell markers in the intact ovaries of different aged mice. (A)** Representative RT-PCR for pluripotent and germ cell markers of 6- and 30-week-old mice is shown. **(B)** The band intensities were normalized to those of GAPDH, respectively and expressed as means ± SD. *P <; 0.05 *vs.* young mice.

The expressions of pluripotent and germ cell markers in the intact ovary, scraped OSE, and postcultured OSE were examined by RT-PCR analyses. As shown in Figure 1, pluripotent markers Oct-4, Sox-2, and Nanog were undetectable in the intact ovary regardless of female age. Whereas, germ cell markers c-Kit, GDF-9, and VASA were significantly decreased in advanced female age (*P <;* 0.05).

**Figure 2:**
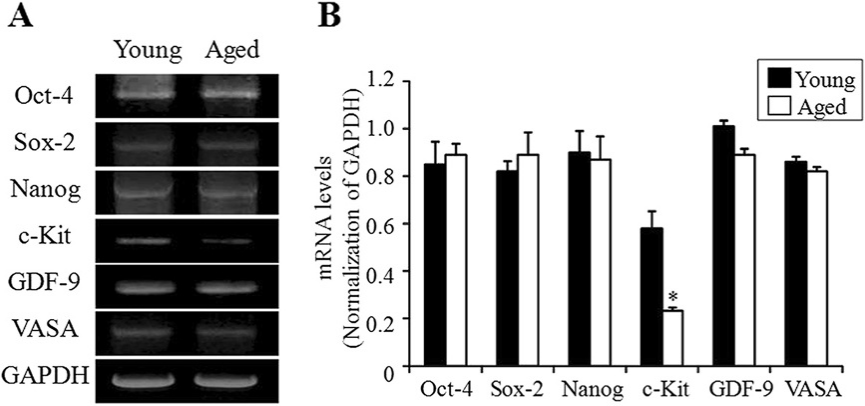
**RT-PCR analysis of pluripotent and germ cell markers in the scraped OSE of different aged mice. (A)** Representative RT-PCR for pluripotent and germ cell markers of 6- and 30-week-old mice is shown. **(B)** The band intensities were normalized to those of GAPDH, respectively and expressed as means ± SD. *P <; 0.01 *vs.* young mice.

All pluripotent and germ cell markers were detected in the scraped OSE, with no significant difference between the two age groups except for c-Kit. The expression of c-Kit was significantly decreased in aged mice compared to young mice (*P <;* 0.01). Additionally, the expression of c-Kit was significantly lower than other pluripotent and germ cell markers (*P* <; 0.05) (Figure 2).

**Figure 3:**
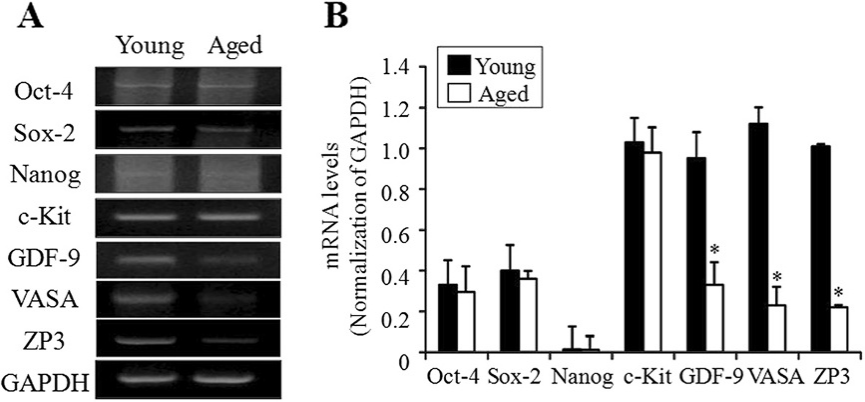
**RT-PCR analysis of pluripotent and germ cell markers in the 3 week postcultured OSE of different aged mice. (A)** Representative RT-PCR for pluripotent and germ cell markers of 6- and 30-week-old mice is shown. **(B)** The band intensities were normalized to those of GAPDH, respectively and were expressed as means ± SD **(B)**. *P <; 0.05 *vs.* young mice.

Transcript for Nanog, which was detected in scraped OSE cells, was undetectable in 3 week cultured OSE; however Oct-4 and Sox-2 were slightly expressed in the postcultured OSE. There were no differences in the expression of all pluripotent stem cells and germ cell markers regardless of female age, except for GDF-9 and VASA, which were significantly decreased in old mice as compared to young mice (*P <;* 0.05). ZP3 was significantly decreased in old mice compared to young mice (Figure 3).

**Figure 4:**
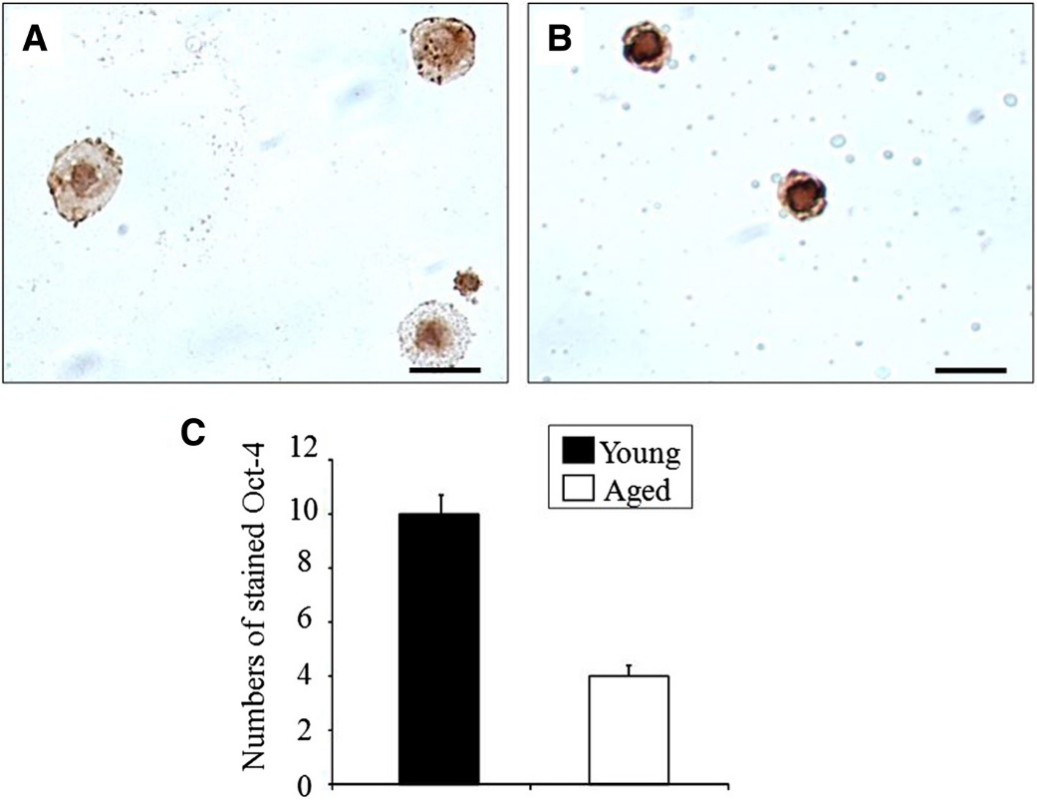
**Immunostaining of Oct-4 in 3-week postcultured OSE of different aged mice.** The oocyte-like structures were developed in 3-week postcultured OSE of young **(A)** and aged mice **(B)** and they were positively stained for Oct-4. **(C)** The numbers of Oct-4-positive cells were counted under a microscope. Scale bar = 20 μm. N=3, 6 ovaries per age group.

The number of OSCs cells was evaluated by immunohistochemical staining for Oct-4 in 3-week postcultured OSE. The number of positively stained Oct-4 was 4 in aged mice and 10 in young mice. The number was 2.5 times higher in young mice, reflecting significant difference between groups (Figure 4).

**Figure 5:**
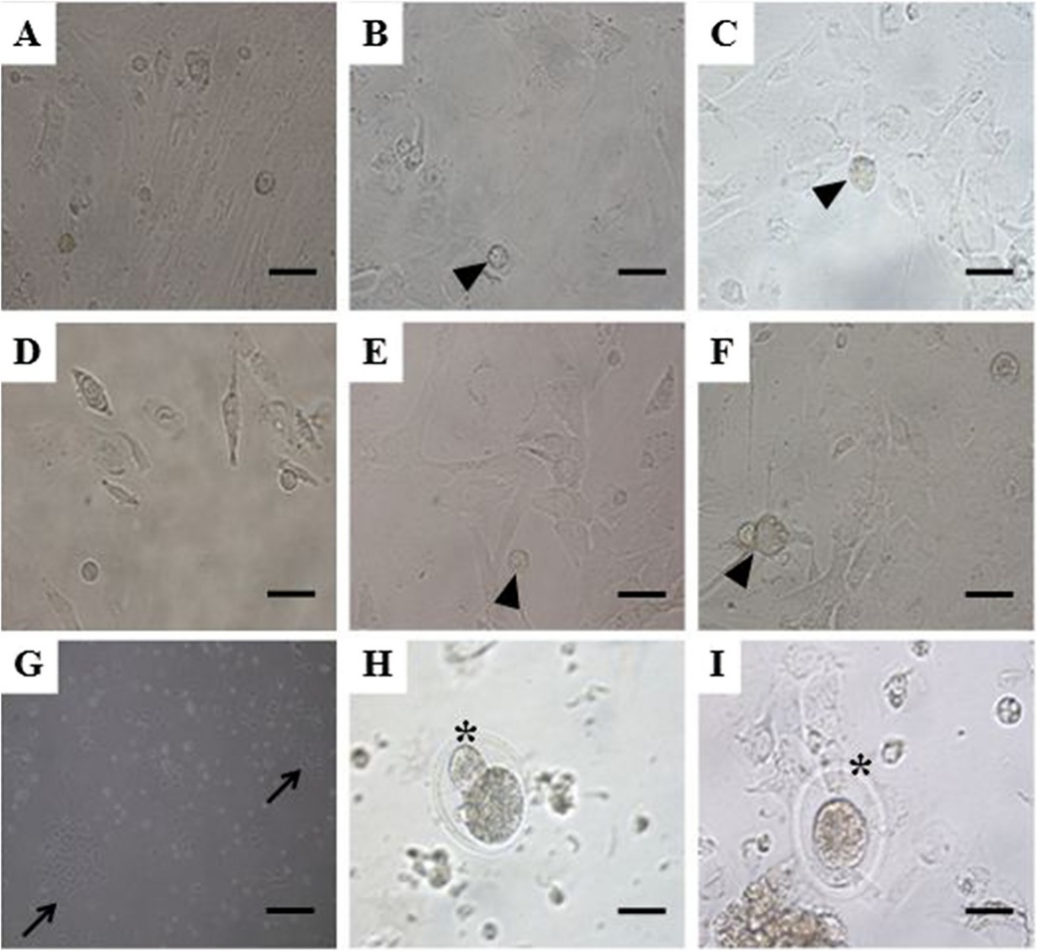
**Smaller oocyte-like structures developed in 3-week postcultured OSE of different aged mice.** Ovarian stem cells spontaneously increased in size and differentiated into oocyte-like structures (arrow head) in 3 weeks postcultured OSE of young **(A-C)** and aged mice **(D-F)**. Single or clusters of epithelial cells (arrow) **(G)** and oocytes (asterix) **(H-I)** were found with OSCs during scraping of OSE. Scale bar = 20 μm.

OSCs spontaneously increased in size and differentiated into oocyte-like structures, regardless of the female age (Figure 5 A-F). Single or clusters of epithelial cells were found at the time of scraping of the ovarian surface, and attached to fibroblast-like appearance cells by postculture processing (Figure 5G). Epithelial cells transformed into spindle-shaped fibroblasts. Small oocyte-like cells resembled bubble-like structure and developed in close proximity fibroblasts by 2 weeks of culture (Figure 5C and F). During initial scraping of ovarian surface tissue, oocytes surrounded by zona pellucida were also collected with OSCs(Figure 5H and I), but they were thoroughly washed together with cellular debris and thus the possibility of occurrence of these oocytes during *in vitro* culture of OSCs was excluded.

**Figure 6:**
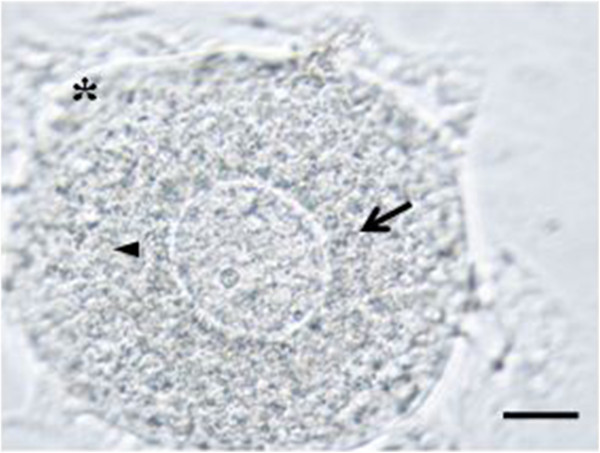
**Larger oocyte-like structures developed in postcultured OSE isolated from young mice.** Larger oocyte-like structures attached to the bottom of the culture dish and showed a prominent nucleus (arrow head), zona pellucida-like structure (arrow) and cytoplasmic organelles (asterix). Scale bar = 20 μm.

Larger oocyte-like structures with prominent nucleus, zona pellucida-like structure and cytoplasmic organelles attached to the bottom of culture dish only in postcultured OSE of young mice (Figure 6).

**Figure 7:**
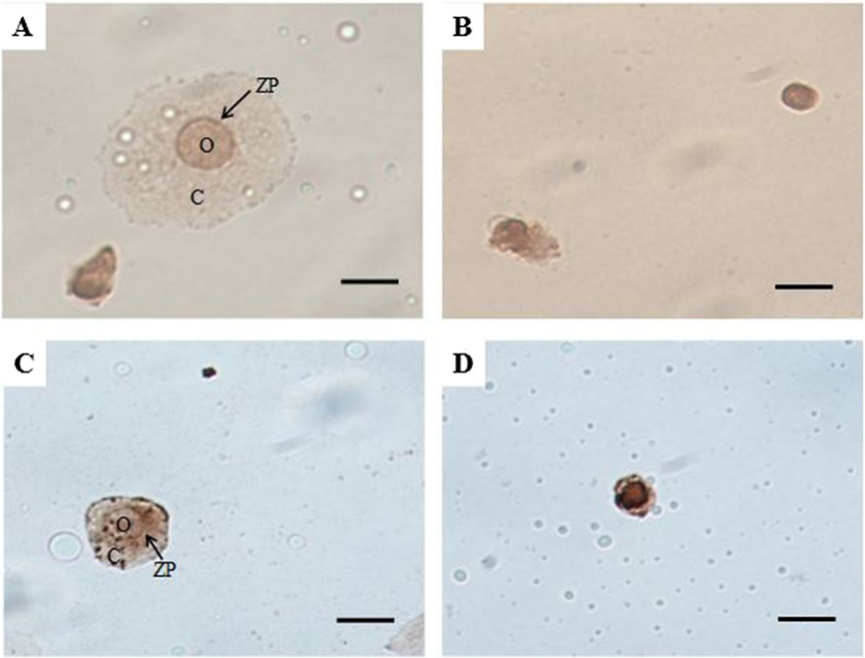
**Immuno-localization of germ cell markers in oocyte-like structures from young mice (A and C) and aged mice (B and D).** The oocyte-like structures stained positive in young mice **(A and C)** and aged mice **(B and D)** for VASA **(A and B)** and GDF-9 **(C and D)** in postcultured OSE. These markers are specific to ooplasm, and surrounding fibroblasts were negative. Oocyte-like structure (O) which surrounded zona pellucid (ZP)-like structure and cumulus **(C)**-like cells is prominently observed in young mice. DAB (3, 3’-diaminobenzidine)-HRP substrate was used to produce a brown reaction in immunohistochemical analysis. Scale bar = 20 μm.

Oocyte-like structures shown in the postcultured OSE were stained positively by immunohistochemistry for VASA (Figure 7A and B) and GDF-9 (Figure 7C and D), regardless of age in female mice. This result indicates that germ cell markers were immuno-localized in oocyte-like structures.

## Discussion

The present study investigated the difference in expression of pluripotent and germ cell markers in the intact ovary, scraped OSE cells, and postcultured OSE cells in female mice according to age. This study showed a reduced expression of germ cell markers, but not pluripotent stem cells markers, in the ovary, scraped and postcultured OSE cells of old females compared to young females. To our acknowledgement, this is the first study to report the differential expressions of germ cell markers in OSE according to female age. This result possibly suggests the decreased potential of differentiation or stemness activity of germ cells in OSE with advancing female age.

Another notable finding in the present study was to show the possibility that pluripotent cells and germ cells presented in scraped OSE cells in aged female as well as young mice and ooycte-like structure was produced from these cells. This result is closely consistent with several previous studies, which showed that OSCs present in aged females can produce oocyte-like structure or oocytes on exposure to an appropriate environment [6, 14, 17–19]. It has been speculated that adult stem cells may play an important role in ovarian function and aging in the female [7].

Some experimental evidence previously indicated that oocyte-like structures were differentiated from the culture of scraped OSEs from postmenopausal women and other adult mammalian species, but only a few of these oocyte-like structures were surrounded by distinct zona-pellucida like structures [8, 11]. We also observed primitive oocyte-like structures in both aged and young mice (Figure 5). However, oocyte-like structure surrounded by distinct zona pellucida-like structure was not detected in aged mice, unlike young mice. The expression of ZP3 was also significantly decreased in old mice compared to young mice. The reason for this different differentiation into oocyte-like structures between young and aged mice is not clear, but may be related to the decreased expression of c-Kit, GDF-9 and VASA as well as ZP3 in aged mice compared to young mice. Decreased expression of germ cell markers possibly indicates the decrease or absence of the differentiation potential of germline stem cells into various cell types. Another possible reason for this decreased differentiation potential into oocytes was recently suggested to be due to the decreased immune activity accompanying age related hormonal changes [20, 21].

Scraped OSEs include epithelial cells, endothelial cells, VSELs, OGSCs, cyst and red blood cells. During the culture of OSEs, VSELs have self-renewal and differentiation potential, and OGSCs proliferate and expand clonally. Usually, several small round cells with a bubble-like structure indicating putative stem cells were observed on day 3 to 4 of culture. In further culture, cell clusters rather than clone was observed. The cell cluster consisted of epithelial cells or spindle-shaped fibroblast and putative stem cells. Previous studies have shown that oocyte-like cells develop with close contact with fibroblast and OSCs [11, 18]. Parte *et al.* suggested that mesenchymal fibroblasts formed supporting granulosa-like somatic cells [11]. These cell clusters were observed in day 6 or 7 cultured OSE, in our study; furthermore, the oocyte-like structures in both age groups of mice appeared to grow in close vicinity to mesenchymal fibroblasts from the cell clusters on day 10 or 11 of culture (Figure 4 C and F). Fibroblasts express aromatase as well as fibroblast growth factor (FGF) [22]. Aromatase catalyzes the turnover of C steroids into estrogens, which play an important role in oocyte development [23, 24]. FGF is involved in cell differentiation, cell migration and angiogenesis [18].

A recent study reported that treatment with FGF and follicle stimulation hormone (FSH) stimulates stem cells present in OSEs and also leads to primordial follicle (PF) assembly [25]. The basic concept of PF development is that the initial development of PF is FSH-independent [26]. However, several previous studies have suggested that PF formation also required the action of FSH [27, 28]. It has been reported that FSH receptors (FSHR) are localized to not only the somatic granulosa cells, but also the normal OSEs [29, 30], ovarian tumor surface epithelium [31], oocytes and cleavage embryos [32, 33]. Since this finding, Demeestere *et al.* showed that FSH possibly coordinates both, the germline and somatic compartments of the mouse follicle [34]. In this respect, it has been assumed that FSH acting through FSHR present in OSE may play a potential role in ovarian biology. Bhartiya *et al*. examined histo-architecture and expression for pluripotent stem cells and germ cell markers in ovaries collected from adult mice during different stages of the estrous cycle, and 2 and 7 days post-eCG (5 IU) treatment to study the effect of gonadotropin on VSELs, OGSCs, postnatal oogenesis and PF assembly. They showed that eCG treatment resulted in increased pluirpotent stem cell activity, neo-oogenesis and PF assembly in adult mouse ovaries. Two days after PMSG treatment, OSE exhibited extensive proliferation [35]. This result is in agreement with earlier reports [36, 37]. Based on these earlier observations, we removed ovaries and collected the OSE 48 hour post-eCG treatment. However, eCG is not equivalent to FSH, since it consists of LH as well as FSH. This study focused on the effect of FSH based on the data of the previous studies. To our knowledge, no studies have reported the effect of LH on pluripotent stem cell activity or OSE proliferation. Therefore, further study on the effect of LH should be done in addition to this research.

Expression of pluripotent (Oct-4, Sox-2, and Nanog) and germ cell (c-Kit, GDF-9, and VASA) markers in the scraped OSEs and postcultured OSEs were observed in previous studies [11, 18]. They showed that the pluripotent markers such as Oct-4, Sox-2, Nanog were strongly expressed in the scraped OSEs, but after *in vitro* culture, putative stem cells strongly expressed Oct-4, and only slightly expressed Sox-2 and Nanog. These findings mean that the scraped OSE had significant and predominant characteristics of pluripotency, and the pluripotency decreased with the culture period. In our study, Nanog was undetectable after 3 weeks of *in vitro* culture of OSE, but expressions of Oct-4 and Sox-2 strongly decreased. Two possible reasons can be considered for the somewhat different expression patterns; firstly species variation between human and mouse, and secondly different sera supplementations in culture media (FBS and fetal calf serum (FCS)). Serum conditions can have a significant effect on stem cell characteristics, such as differentiation and proliferative capacity, as shown for the proliferation and osteogenic differentiation capacity of human adipose stem cell, wherein a notable difference was found in collagen type I and ALP mRNA expression [38].

It is possible that OSCs from different aged mice express different levels of the same pluripotent markers. Thus, the expression level of these markers may not reflect the number of OSCs in the OSEs samples. Therefore, in this study we performed immunohistochemical staining of Oct-4 (pluripotent marker) in the postcultured OSEs and showed a significantly higher number of Oct-4 positive cells in young mice than aged mice. This result did not coincide with the results from RT-PCR analyses of postcultured OSEs, which showed no significant difference between the age groups. The reason for different results between immunohistochemical staining and RT-PCR is not clear. However, it was likely that cells collected for RT-PCR may have contained fibroblast cells, as well as oocyte-like structures because oocyte-like structure developed in close contact with fibroblast cells.

Recently, Oct-4A is an nuclear Oct-4 and has become a known marker for the pluripotent state. During differentiation, nuclear Oct-4A shifts to the cytoplasmic Oct-4B. VSELs express Oct-4A and OGSCs express Oct-4B. Oct-4A is amplified by Oct-4A primer whereas Oct-4B is amplified by Oct-4 primer which amplifies all isoforms [39]. Oct-4A must be appropriate if we want to examine pluripotency of OSE or OSCs. However, this study focused on the germ cell activity as well as pluripotency of OSE, hence the use of Oct-4 was more appropriate than Oct-4A.

Unexpectedly, the expression of pluripotent markers was undetectable in the intact ovary whereas they could be detected in OSEs. Ovarian cortical tissue showed minimal OSEs and few primordial follicles [25] and ovaries of reproductive-age women possess rare mitotically active germ cells [15]. Therefore, it seems that the level of the expression of pluripotent markers in the intact ovary may be relatively too small to be detected by RT-PCR as compared to those of concentrated OSEs. Contrastingly, mouse ovaries have many germline stem cells from 2 distinct populations with different diameters: cells with diameters of 10–15 μm in the ovarian surface epithelium and cells with diameters of 50–60 μm in the center of the follicular compartment [40]. Therefore, the expression of germ cell markers for c-Kit, GDF-9, and VASA in the intact ovary could be observed.

Real-time PCR has been recently used for quantitative evaluation of gene expression, but this study evaluated quantitatively gene expression of each marker using Image J. For the past 25 years, NIH Image and Image J software have been widely accepted as a semi-quantitative evaluation method of gene expression [41] by normalizing the relative band intensities of target gene expression obtained in the RT-PCR assay to those of GAPDH.

Expression of GDF-9 and VASA, among germ cell-specific markers, in postcultured OSEs were significantly decreased in old mice compared to young mice. GDF-9 is among the key oocyte-secreted factors (OSFs) and can activate signaling pathways in cumulus cells to mediate the development of neighboring oocytes and play vital roles in oocyte maturation and quality determination [42, 43]. Aged oocytes had a decreased expression of GDP-9 [44]. Mouse vasa homologue (MVH) is expressed exclusively in the ovary and has been characterized as a marker of primordial germ cell lineage, such as the early stages of germ cell differentiation.

Unlike the expression pattern of GDF-9 and VASA, the present study showed decreased expression of c-Kit in the scraped OSE, but not in postcultured OSE, in aged mice compared to young mice. The reasons for this difference in gene expression were possibly 2 fold: Firstly, the technical error in the experimental procedures. However, we ruled out this possibility based on reproducibility of the data on repeated experiments. The other possible reason was that these markers were detected in different periods of differentiation. Usually c-Kit is a pre-differentiation marker, whereas VASA and GDF-9 are post-differentiation markers [45]. Several studies have shown that the cultured OSEs express high levels of c-kit receptor and its ligand SCF proteins *in vitro*[18, 46]. This result was also observed in postcultured OSE in the present study.

Mice typically breed from 6 to 8 weeks of age and continue for about 200 days depending on the strain [47]. The sexual maturity and lifespan in laboratory mice is around 6 weeks and 1 year, respectively [48]. Considering the reproductive physiology of mice, 6 to 9 weeks, 14 to 16 weeks, and 25 to 27 weeks in mice may be comparable with teenage years, ≥30 years of age, and ≥40 years of age in humans, respectively. Niikura *et al.* considered mice of 20 month old as aged [14]. Twenty month old mice may be very old with complete loss of reproductive function, comparable with around 60 years of age in the human. Furthermore, oocytes collected from 30-to-40-week-old mice are more developmentally sensitive to mitochondrial damage than oocytes from pubertal mice, resulting in significant decline of oocyte competence [49]. Therefore, 20 month old mice are preagonal and seem to be too old to evaluate the status on the reproductive physiology. In this respect, we thought that 28–30 week old mice used in our present study were an adequate model of human menopause and/or pre-menopause.

## Conclusions

This study demonstrated that the expression of germ cell markers, but not pluripotent stem cells markers, in the intact ovary and postcultured OSE cells of old females may be decreased compared to young females. Additionally, this study showed that OSE-derived OSCs produced oocyte-like structure even in aged mice, although the oocyte-like structures in aged mice were incomplete as compared to young mice. These results suggested that advancing female age resulted in decreased potential of differentiation into oocytes or stemness activity of germ cells in OSEs. Therefore, further study is required to understand the appropriate milieu for inducing regeneration of oocytes from OSCs in aged female. It is expected that this study may contribute to the development of a new strategy for the production of oocytes in the treatment of female age-related infertility and POF.

## Abbreviations

eCG: Equine chorionic gonadotropin
OGSCs: Ovarian germ stem cells
OSCs: Putative ovarian stem cells
OSE: Ovarian surface epithelium
POF: Premenopause ovarian failure
RT-PCR: Reverse-transcription polymerase chain reaction
VSELs: Very small embryonic like stem cells.
